# IoT Clusters for Enhancing Multimedia Applications

**DOI:** 10.3390/s22239077

**Published:** 2022-11-23

**Authors:** Jorge Coelho, Luís Nogueira

**Affiliations:** 1 School of Engineering (ISEP), Polytechnic of Porto (IPP), 4249-015 Porto, Portugal; 2 Artificial Intelligence and Computer Science Laboratory, University of Porto (LIACC), 4099-002 Porto, Portugal

**Keywords:** IoT, clustering, QoS assurance, resource usage optimization, edge computing, computational offloading

## Abstract

In this paper, we present a framework for exploring the spare capacity of IoT devices for clustered execution of multimedia applications. Applications of this type are usually framed with specific quality parameters that enable a desirable level of service. This means that the IoT cluster must guarantee strict quality ranges of service to work as expected. The framework is totally customizable, and QoS dimensions can be easily added or removed given their relevance in the application scenario. The achieved results clearly demonstrate the utility of using the spare capacity of IoT devices, otherwise unused, to cooperatively execute servies within the desired quality of service levels.

## 1. Introduction

Internet of Things (IoT) devices are becoming ubiquitous due to their quantity and proliferation [1,2,3]. This means that there is a considerable amount of computational power distributed by these devices. It has been our goal to explore the use of these devices, particularly through the creation of tools that enable code distribution and execution to explore the use of the spare computational capacity of IoT devices [4,5].

The single-board computer (SBC), a rather powerful type of machine that can be used as a generic IoT device, enjoys enormous popularity due to their high performance for their price range and the vast number of settings wherin they can be used. These devices are becoming a standard for IoT prototyping and implementation [6,7,8], bringing more and more computing power to this domain and also more underutilized devices.

Our goal is to create tools to enable the use of the spare power of such devices to accomplish tasks in collaborative scenarios were parallelization is key. One scenario where we believe that a coalition of IoT devices can make a difference is multimedia-based applications. Due to their nature, multimedia applications, such as the ones that process video or audio, benefit from the parallelization of hardware, as many tasks are extremely parallelizable [9,10,11]. At the same time, they are also associated with QoS constraints that bring a new challenges to the implementation of distributed service execution. These constrains must be fulfilled, and the coalition of IoT devices must guarantee that it is possible to deliver such service.

In our scenario, IoT devices have different applications that are already being executed. The idea is to use their otherwise unused spare capacity. This means that we need to be able to measure the amount of spare capacity each device has in order to use it without interfering with its normal function.

It is well known that resource allocation in large distributed systems is an NP-hard problem. Due to the complexity and dynamism of applications, it is difficult to foresee the amount of load that will be imposed. Therefore, static allocation tends to create underutilized platforms due to used worst-case resource reservations. To achieve our goals, a dynamic approach which relies on lightweight heuristics that can be dynamically applied during runtime is the obvious solution. One can find a comprehensive survey of the kinds of resource allocation heuristics that can cover different levels of dynamicity while coping with the scale and complexity of high-density many-core platforms in [12]. Here, we apply such concepts in order to know the amounts of resources (such as processor cycles, communication bandwidth, disk bandwidth, and storage) each device can contribute to a coalition of IoT devices and create a platform for distributed multimedia computations. Similar approaches to other domains can be found, for example, in [13,14].

Streaming consists of the continuous transmission of video and audio instead of downloading all the data first. Streaming protocols have been studied for a long time, and there is a reasonable amount of work about applying streaming protocols to IoT devices [15,16,17]. Our purpose here is not to define how the streaming of data occurs at its core, but to describe the creation and coordination of IoT clusters to deliver services that can use streaming protocols, guaranteeing some expected quality of service.

Clusters of IoT devices were the topic of several previous works. In [18], the authors propose a distributed architecture to perform collaborative work for IoT environments and sharing the application workload among the available devices. Offloading of tasks has been the subject of several previous works. In [19], the authors proposed an algorithm to provide efficient task offloading in IoT and fog computing nodes. In [20], the authors develop a distributed task algorithm in the context of fog nodes, where the spare resources are also a relevant feature for the problem solution and are provided by what the authors call helper nodes. A two layer architecture that includes one layer with clusters of IoT devices performing several tasks is presented in [21]. Sharing of unused computational resources of IoT devices in a clustered approach in the context of smart cities was the subject of [22]. The efficiency of IoT devices in the context of healthcare is studied in [23,24], where the authors apply task level parallelism (TLP) as a technique to optimize resources. This technique is also used at the device level in our approach as a further optimization technique. Using offloading solutions to improve efficient computation in IoT devices for other domains of application can be found, for example, in [25,26,27,28]. Although there are several previous work on this subject, to the best of our knowledge, the work we present here is the first one using IoT devices in collaboration to process multimedia applications.

The remainder of this paper is organized as follows. In the next section, we present definitions and details needed to describe our framework; then we present the core algorithms, along with examples of their application. We then validate our approach by showing its application to real and simulated scenarios and by analyzing the results. Finally, we conclude and present future work.

## 2. System Model

Our approach consists of a cluster of IoT devices that deliver some service, guaranteeing predefined QoS constraints. In this section, we describe, by means of definitions and examples, the several components of the system and how they interact.

**Figure 1:**
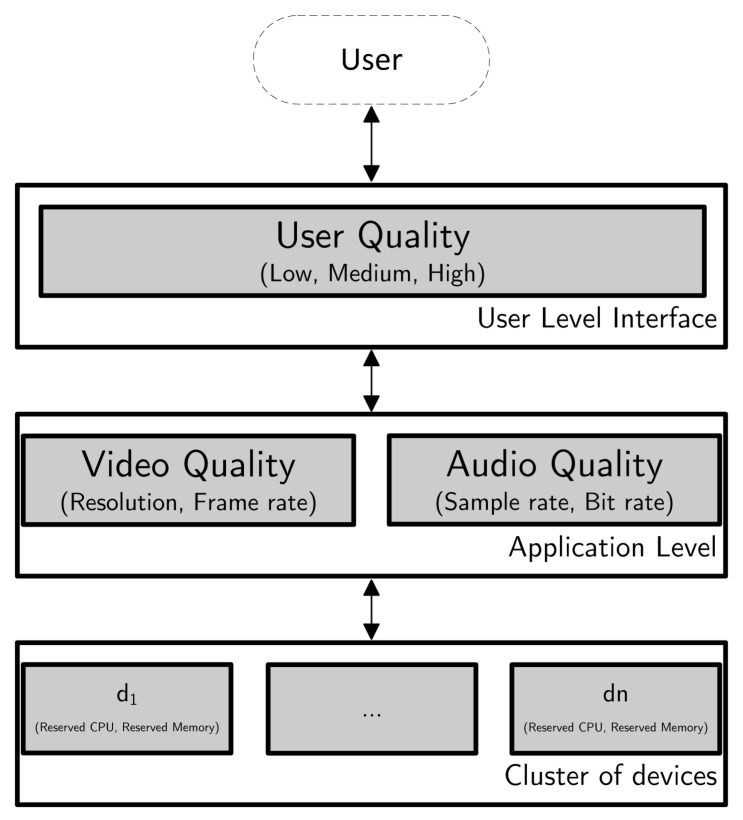
High-level view of the system.

We start by presenting, in Figure 1, a high level view of the system as a whole, where the user defines the quality needed for a service, which is then translated in application properties and finally is executed by the cluster of devices that use a reserved portion of resources to deliver the service with the adequate QoS to fulfill the user’s request.

Code distribution is the central feature of this system. IoT devices with spare capacity offer their availability to participate in a coalition in order to allow the system to achieve a global outcome in terms of service delivery with a given quality. Due to the ad hoc nature of this coalition, several details must be thoroughly studied, namely, how to communicate, distribute data and guarantee the global QoS, and how to deal with dynamic changes.

Resource reservation plays an important role due to establishing the exact spare capacity that the device is willing to concede to a coalition, assuming such guarantee is central to enable the coalition to properly work. However, only this will not guarantee that the provided QoS is stable, since new devices can be added and removed (due to failures) at any time. Therefore, during runtime, the system must assure that a given QoS level is still possible, or a change to a new degraded (when acceptable) version of the service will be chosen. Whenever that option is not possible, the coalition will not be able to continue to provide service.

We now present several definitions.

**Definition** **1** (IoT Cluster). *An IoT cluster is a set $C=\{d_{1},\ldots ,d_{n}\}$ of IoT devices $d_{i}$ that are currently providing a collaborative solution for a service.*

**Definition** **2** (IoT Sub-cluster). *Given an IoT cluster $C=\{d_{1},\ldots ,d_{n}\}$, a sub-cluster $S_{C}$ is a set $\left\{d_{i1},\ldots ,d_{in}\right\}$ of IoT devices where each $d_{ik}\in C$.*

**Definition** **3** (Processing Unit). *A processing unit p is defined as {t,u}, where t is a task of code that should execute with input data u.*

**Definition** **4** (Service). *A service S={P,Q} is a set $P=\{u_{1},\ldots ,u_{n}\}$ of processing units along with QoS constraints defined in Q.*

A service can be provided with different QoS levels due to the nature of the service and the user’s quality preferences.

**Definition** **5** (QoS Constraints). *Let Q be the set of the user’s QoS constraints associated with service S. Each $Q_{kj}$ is a finite set of quality choices for the $j^{th}$ attribute of dimension k. This can be either a discrete or a continuous set such that Q={Dim,Attr,Val,DAr,AVr,Deps}, where Dim is the set of QoS dimensions, Attr is the set of attributes identifiers and Val is the set of attributes’ values identifiers. Each value is represented by a tuple $Val_{i}=\left\{Type,Domain\right\}$, where Type={integer,float,string}, and Domain={continuous,discrete}.*

*The set of relationships $DA_{r}$ assigns to each dimension in Dim a set of attributes in Attr and is defined as $DA_{r}:Dim_{i}\to Atr,\forall_{Dim_{i}}\in Dim$.*

*The set of relationships $AV_{r}$ assigns to each attribute in Attr a specific value in Val and is represented as $AV_{r}:Atr_{i}\to Val_{k},\forall_{Atr_{i}}\in Atr,{\exists^{1}}_{Val_{k}}\in Val$.*

*Deps defines the set of existing dependencies among the values of the existing attributes. A dependence between $Atr_{i}$ and $Atr_{j}$ is represented as $Dep_{ij}=f\left(Val_{ki},Val_{kj}\right),\forall Attr_{i},Attr_{j}\in Attr$.*

For a given service, one can choose from a set of values for QoS dimensions; for example, audio-related parameters such as the sampling rate (8, 16, 24, 44, 48 and 88 kHz), sampling bits (8, 16 and 32), end-to-end latency (100, 75, 50 and 25 ms) and video related parameters such as picture resolution (SQCIF, QCIF, CIF, 4CIF, 16CIF), color depth (1, 3, 8, 16, *…*) and frame rate (1, *…*, 60).

Users provide a specification for the minimum desired QoS for a service *S* with the minimum acceptable dimensions. Delivering the service can be done with values that meet at least those defined by the user.

**Example** **1.** *Using a video streaming application as an example, the following is a list of quality dimensions that might be associated with any particular application. The list is given to illustrate the proposed model and is not intended to be exhaustive.*

*Dim = {Video Quality, Audio Quality}*
*Attr = {compression index, color depth, frame size, frame rate, sampling rate, sample bits}*
*Val = {{1, integer, discrete},{3, integer, discrete}, …, {[1, 30], integer, continuous}, …}*
*DA Video Quality = {image quality, color depth, frame size, frame rate}*
*DA Audio Quality = {sampling rate, sample bits}*
*AV compression index = {[0, 100]},*
*AV frame size = {SQCIF, QCIF, CIF, 4CIF, 16CIF}*
*AV color depth (bits) = {1, 3, 8, 16, 24, …}*
*AV frame rate (per second) = {[1, 30]}*
*AV sampling rate (kHz) = {8, 11, 32, 44, 88}*
*AV sample bits (bits) = {4, 8, 16, 24}*

*Having such a QoS characterization of a particular application domain, users and service providers are now able to define service requirements and proposals in order to reach an agreement on service delivery. Since the QoS has a multi-dimensional nature, tradeoffs can be made due to the scarcity of resources.*

Further details on QoS characterization for distributed systems can be found, for example, in [13].

From a pragmatic perspective, one can hide the details of QoS characterization in high-level descriptions, as presented in the following example.

**Example** **2.** *From a user’s perspective, and for practicality, QoS dimensions can be simplified. For example, video can be simply described as SD, HD and FHD, as presented in Table 1; and audio as low, medium and high, as presented in Table 2.*

*Please note that this is just an example and other configurations can be easily implemented.*

**Table 1:** High-level video definitions.

Description	Common Name	Resolution
SD (Standard Definition)	480 p	640 × 480
HD (High Definition)	720 p	1280 × 720
FHD (Full HD)	1080 p	1920 × 1080

**Table 2:** High-level audio definitions.

Resolution	Bit Rate (kbps)	Sample Rate (kHz)
Low	128	32
Medium	192	44.1
High	320	48

Moreover, note that QoS constraints are defined as the minimum acceptable set of properties to deliver a given service. This means that the coalition can produce a higher quality outcome, but not a lower one. Thus, devices leaving the coalition (due to some type of failure) must be handled, and the IoT cluster’s minimum QoS delivery must be recalculated.

### 2.1. Algorithms

The framework is divided in two main modules, the cluster setup and dynamic adaptation to new services. IoT clusters collaborate in order to fulfill the requested service by processing data framed with the QoS defined by the user. Due to the heterogeneity of services to be executed, users’ quality preferences, underlying operating systems, networks and devices, QoS specification must be acknowledged by all the devices, or they must be able to map their individual specifications onto a common one.

It is important to note that although we do not explore streaming algorithms, we need to understand the capabilities of the different devices in our cluster. Thus, we estimated their processing power in order to conclude if they are able to cope with the imposed demand.

#### 2.1.1. Cluster Setup

The first step to have a collaborative network of IoT devices working together to deliver a multimedia service is to know how many devices are available and what they are capable of, and therefore, estimating the global power at hand. Cluster formation consists of registering available IoT devices with the main device (the one who is requesting the service) and announcing the spare capacity to deliver. Since the goal is to process audio and video, each device announces its capacity in a worst-case scenario with the spare resources it has. This is then mapped with the user’s preferences, and if it is possible, the service is delivered. Each IoT device registers with the device that is requesting to run the service in a client–server model. In Algorithm 1, we describe how this process works.
**Algorithm 1** Cluster setup.
Let N be the node requesting the service *S*.Let A:={} be a global variable that stores the set of available nodes in the cluster. Take a service S={P,Q} with processing unit *P* and related QoS *Q* such that each $Q_{kj}$ is a finite set of *n* quality choices for the $j^{th}$ attribute, expressed in decreasing order of preference, for all *k* QoS dimensions. Let S:={} be the set of nodes in the cluster capable of providing a given service. Let N broadcast to the local network the request for nodes to register, adding them to A.  1: **for** each $d_{i}\in A$ **do**
 2:       Let $Q_{j}$ be the QoS delivered by node $d_{i}$
 3:       **if** $Q_{j}$ is higher (in all its dimensions) than *Q* **then**
 4:            $C=C\{\left(d_{i},Q_{j}\right)\}$ 5:       **end if**
 6: **end for** 7: **return**
C


#### 2.1.2. Dynamic Adaptation to New Services

In Algorithm 2, we present the coordination for accepting new services.
**Algorithm 2** Dynamic adaptation to new services.Let N be the node requesting the service $S_{new}$.Given a new service $S_{new}=\left\{P_{new},Q_{new}\right\}$ with processing unit $P_{new}$ and related QoS $Q_{new}$ such that each $Q_{kj}$ is a finite set of *n* quality choices for the $j^{th}$ attribute, expressed in decreasing order of preference, for all *k* QoS dimensions. Let A be a global variable that stores the set of available nodes in the cluster.Let $S_{C}=\left\{\right\}$ be the sub cluster that will provide the new service.Let N broadcast to the local network the request for nodes to register adding them to A. **while** t < timeout **do**       **for** each $d_{i}\in A$ **do**             Let $Q_{j}$ be the QoS delivered by node $d_{i}$             **if** $Q_{j}$ is higher (in all its dimensions) than *Q* **then**                   **if** $d_{i}$ can accommodate such service along existing ones **then**                         $S_{C}=S_{C}\cup \left\{d_{i}\right\}$                   **end if**             **end if**        **end for** **end while** **return**
$S_{C}$


Note that services can be added up to a number where they can be delivered. In the case of failure of one node, the service is reset and a new sub-cluster is defined.

**Figure 2:**
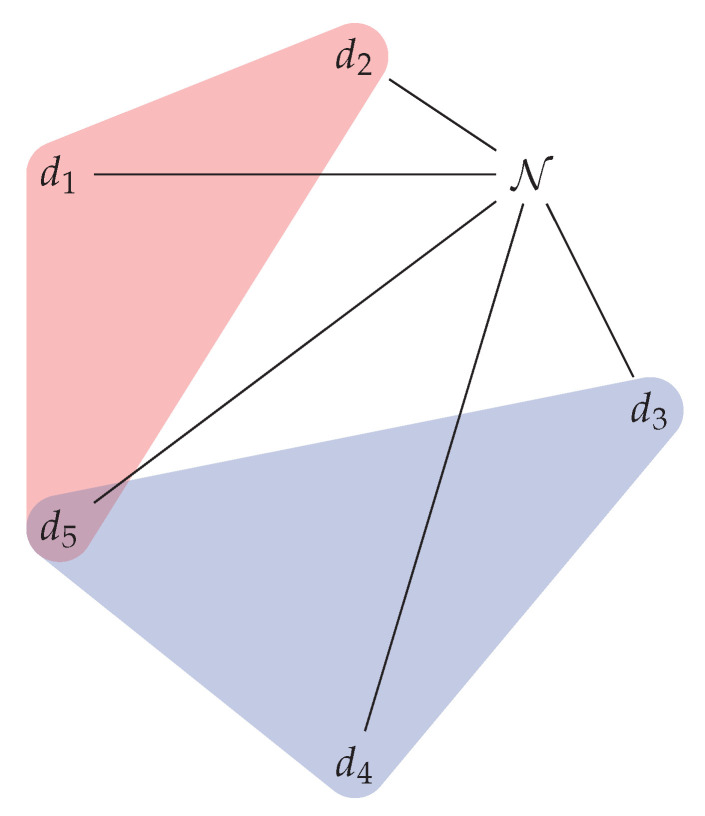
Two sub-clusters providing different services $S_{1}$ and $S_{2}$ with different QoS.

**Example** **3.** *In Figure 2, we can see two sub-clusters that were created to provide two different services with different QoS. Device N requested a first service $S_{1}$ that was provided by devices $\left\{d_{1},d_{2},d_{5}\right\}$, and after this, a second service $S_{2}$ that was provided by devices $\left\{d_{3},d_{4},d_{5}\right\}$. Both services are based on the available devices that are part of the main cluster ($\left\{d_{1},d_{2},d_{3},d_{4},d_{5}\right\}$). This cluster is dynamic, since it changes by adding and removing devices which can occur at any time.*

All the algorithms presented here are high-level descriptions of the implementation; many minor details are not described. In the next section, we look deeper at how the actual framework works by describing relevant parts of the implementation.

## 3. Results and Analysis

For the implementation of the framework, we used Elixir programming language [29] because of the ease it provides to distribute and execute data and code by devices in a network. For the hardware, we used Raspberry Pis, a type of SBC (single-board computer) that can be used as a generic IoT device and enjoys enormous popularity due to is high performance for its price range and the vast number of scenarios where it can be used [6,7,8]. The use of this type-o SBC also allows the use of Linux as the operating system to implement a resource reservation policy and use Elixir along with the Erlang virtual machine [30] in each one of them, creating a distributed scenario that is simpler to manage.

### 3.1. Cluster Formation

**Figure 3:**
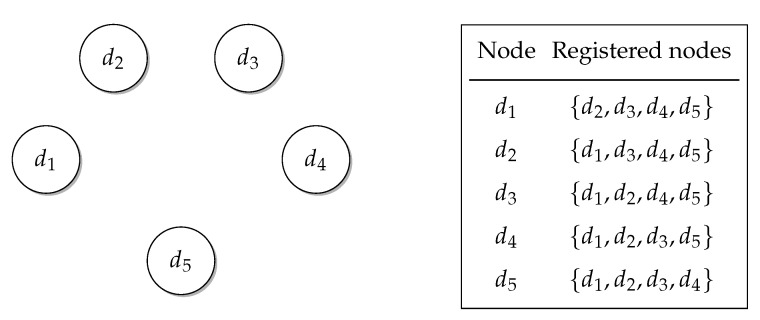
Node discovery.

All the IoT devices ran a client–server program that allowed them to interact with others and enabled the creation of the cluster that would provide the service. Any IoT device when turned on announces it is available. This is done by using built-in discovery features of Elixir, and every device maintains a list of all the other known devices in the network, as shown in Figure 3.

**Figure 4:**
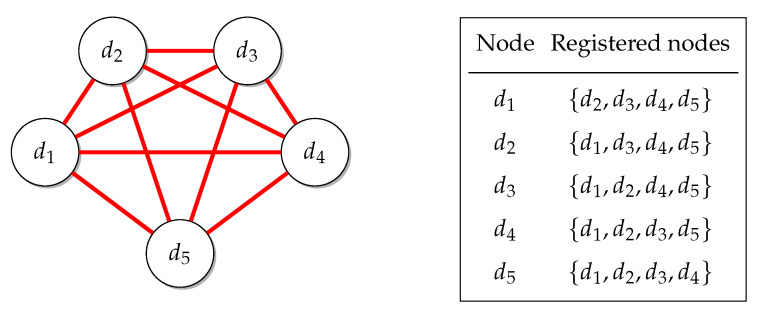
Node linking.

**Figure 5:**
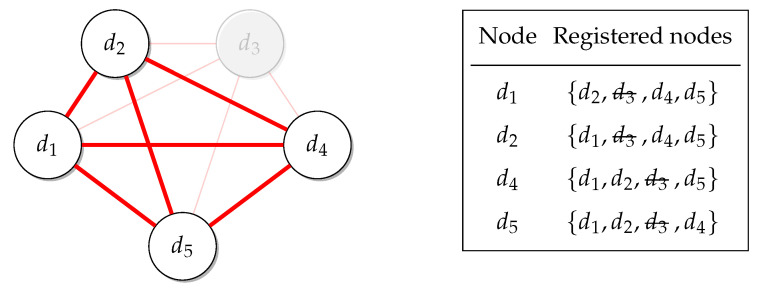
Failure of node $d_{3}$.

We say that these other devices are registered with the device that has them on its list. The framework also provides a feature in which a link between devices is maintained, as seen in Figure 4. Any failure (device disconnecting) is then detected, allowing the list to be updated by removing the disconnected device, as seen in Figure 5.

When one of these devices needs to run a service, it queries each of the devices that are registered with it, obtaining the QoS capability that each can provide. The devices that provide a QoS above the minimum are selected to collaborate. As an example, we present in Listing 1 a small example written in Elixir of the main function of the cluster formation.


		Listing 1: Cluster formation main function.
           def create_cluster(RegistredNodes,QoSParametersList) do
               SubCluster = query_nodes(RegistredNodes,QoSParametersList)
           end 
		    
           def query_nodes([],[]) do
               []  
           end
		   
           def query_nodes([node|remainingnodes],qosparameterslist) do 
               send(node, {:evaluate,qosparameterslist}
               receive do
		  {node,:ok_capable} ->
                      [node|query_nodes(remainingnodes,qosparameterslist)]
		  {node,:not_capable} ->      
	              query_nodes(remainingnodes,qosparameterslist)
	       end
	   end
		   

### 3.2. Local QoS Calculation

A local QoS calculation allows one to know if a node can collaborate in a service given specific QoS constraints. This needs to be fast in order to cope with the dynamism of the framework. Our proposed solution for this specific problem is to benchmark the devices and know their capabilities in advance. Since we were using SBCs, we could profile those devices in order to know how much CPU and memory is needed to deliver some service under specific circumstances. We benchmarked all the SBCs that are used in the framework and obtained their resource needs for processing video and audio under some typical scenarios that we defined. Again, the framework can be modeled with different SBCs and different configurations of video and audio streams, and this should be seen as the example which we use in our solution, but that can be easily adapted to other situations.

We used the *ffmpeg* tool (https://ffmpeg.org/) (accessed on 4 November 2022) and the resource usage monitoring utility *RPI-Monitor* (https://github.com/XavierBerger/RPi-Monitor) (accessed on 4 November 2022) to test and obtain statistical data on typical scenarios for the SBCs considered, namely, the Raspberry Pi 3 A+, 3 B+ and Zero W.

We measured CPU and memory load for decoding video and audio in the following scenarios:

- Low—SD video and medium audio quality: Video resolution of 640 × 480 pixels with 24 frames per second and audio with 128 kbps of bit rate and 32,000 Hz sample rate.
- Medium—HD video and high audio quality: Video resolution of 1280 × 720 pixels with 24 frames per second and audio with 192 kbps of bit rate and 44,100 Hz sample rate.
- High—FHD video and very high audio quality: Video resolution of 1920 × 1080 pixels with 30 frames per second and audio with 320 kbps of bit rate and 48,000 Hz sample rate.

**Figure 6:**
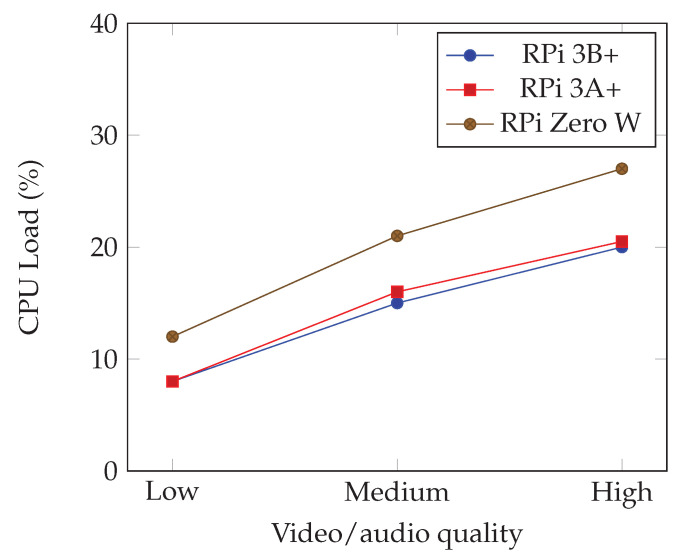
Processing power needed.

The CPU load is presented in Figure 6, where one can see that among the models Raspberry Pi 3B+ and 3A+, there was almost no difference in the CPU load. This was expected, as they rely on the same hardware at the CPU level. The Raspberry Pi Zero W has a less powerful processor, and this is clearly noticeable. While the CPU was stable across all our tests, we noted that this was not the case for RAM, where several configurations of the decoder would lead to rather different RAM requirements. In this case, we noticed that it needed an average of 27 MB for decoding the lowest quality stream, 45 MB for the medium quality stream and 56 MB on average for the highest quality one; and this applies to all the devices we tested.

Please note that in these tests we did not use H.264 hardware acceleration, as not all the devices provide this feature. Therefore, we directed our approach to the typical IoT device that relies mainly on the CPU’s capability to process all the work. Nevertheless, we can say that with H.264 enabled, the Raspberry Pi 3 had a constant CPU load of 5% for all the different combinations of video/audio tested and a constant memory footprint of 22 MB, making it a strong candidate for enabling fast and efficient processing of audio and video streams, which we hope to explore in future work.

Finally, we reinforce that this is a configuration step of the framework, and any other values and dimensions can be considering when the setup phase occurs. Additionally, we tested stream encoding and transcoding, but we verified that this is a much more intensive task and that the hardware at hand could not cope with the demand for rather low-quality streams.

### 3.3. Dynamic Coordination

When a new service arrives, a QoS request, is performed for the registered devices, and the computation is done as explained in Section 3.2. If it is possible to provide the service, then a new sub-cluster is created for this service following the procedure described in Algorithm 2. All the coordination relies on the the Elixir message system.

When one device fails, the framework detects that event immediately, as illustrated in Figure 5. After studying several approaches, we concluded that the most adequate one is to reset the cluster whenever there is a node failure. This means that the whole process of querying registered nodes and forming the service providing coalition is done again. Since this process relies on local network communication with very low latency and that the data used to setup the configuration are available statically, this process is relatively fast. This means that, in the case, where there is still a possibility to proceed with the service, the user will be deprived of it just briefly.

## 4. Validation

In this section, we analyze how our approach performs in terms of cluster setup and resource management. We used a physical cluster for preliminary tests in which different services were requested and the needed resources were reserved. Then, we created a simulation that enabled the testing of a larger number of devices and hardware configurations and analyzed how resources are used under our approach.

### 4.1. Simple Hardware Cluster

For our first scenario, we used the hardware described in Table 3, with five IoT devices and their respective available CPU and memory, after a resource reservation policy was applied.

We configured several simultaneous services. One example is presented in Table 4.

Device $d_{1}$ has all the others ($d_{2}$, $d_{3}$, $d_{4}$, $d_{5}$) in its list of registered nodes and $d_{2}$ has ($d_{1}$,$d_{3}$, $d_{4}$, $d_{5}$). In Table 5, one can see what happens after adding services $S_{1}$, $S_{2}$ and $S_{3}$ to the cluster. After service request $S_{1}$, all devices except $d_{5}$ are able to collaborate, allocating the resources defined in Section 3.2 to participate. When service $S_{2}$ is added to the cluster, all devices except $d_{3}$ can participate in the associated sub cluster. Note that, since the resources needed are less than the ones needed by $S_{1}$, now device $d_{5}$ is also able to participate. Finally, adding $S_{3}$ sees the collaboration of $d_{2}$ and $d_{4}$. When testing, we noted that using the CPU to its limit is not a good idea due to slight variations of CPU load during executing, which in case of an overloaded CPU can delay the execution of the assigned services; thus, we believe that leaving a small proportion of the CPU always available is a good idea (typically at least 5%).

### 4.2. Simulation of Bigger Clusters

After conducting our first experiments with real hardware, and since statistical data estimated the average number of connected devices per household in 2023 as 13 in North America and 9 in Europe [31], we decided to use a simulator in order to escalate the size of the cluster and test with different configurations. We implemented these simulations in Elixir using Erlang Virtual Machine processes. Devices are simulated by processes where each one maintains a list with total and remaining CPU and memory. Communication is simulated by using the internal process communication features, and latency is ignored. The types of services that can be bind to devices follow the ones presented in previous sections, and there is a profile telling what they consume in terms of CPU and memory. For this simulation, we used the characterization described in Table 6.

With this simulation, we can create as many processes and as many services as we want, given that we do not exceed the underlying virtual machine limits. However, the idea was to try to match what could possibly be found in a real scenario. For example, with 15 devices and several services running, one could generate the cluster including the devices described in Table 7, where for each device we also detail the reported available RAM in number of megabytes and the available CPU in percentage.

We can also generate the list of services described in Table 8.

**Figure 7:**
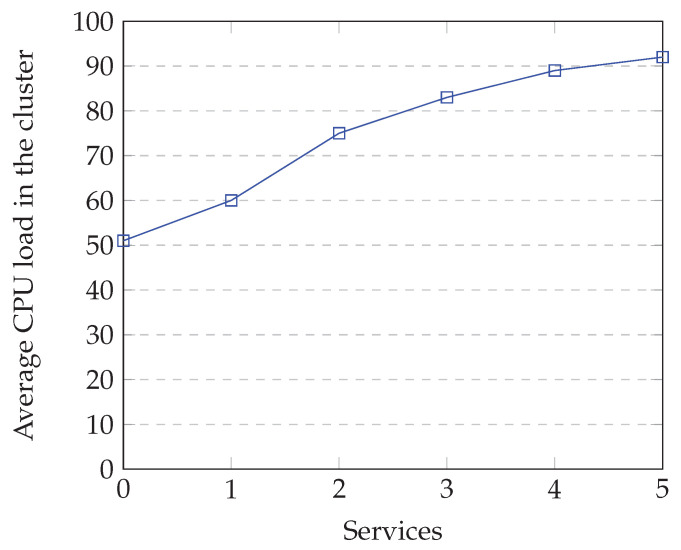
Average CPU load in the cluster upon introduction of services.

**Figure 8:**
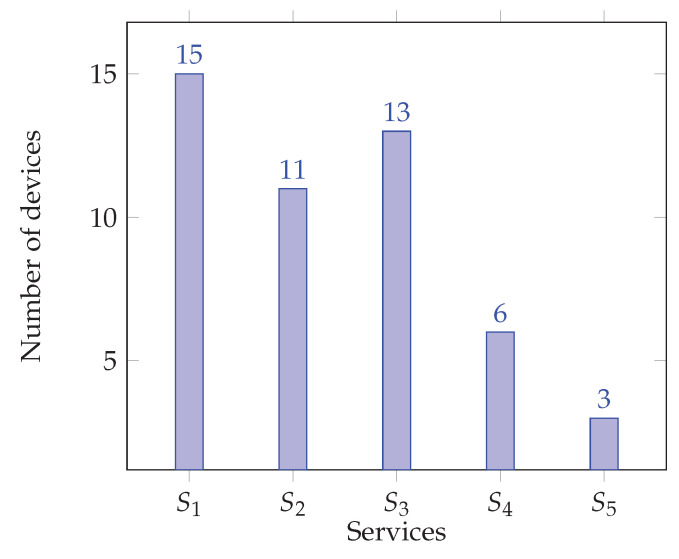
Number of devices participating in sub-clusters.

The optimization of the resources depends highly on the order of arrival of the services, but for the previous scenario, one can note a considerable increase resource usage. In Figure 7, one can see the amount of average CPU power used in the cluster as the services are introduced. In Figure 8, one can see how many devices are able to collaborate in the execution of the services. The number of devices decreases with the introduction of the services, since some run out of resources (mainly CPU power) and are unable to participate in the collaborative effort.

Please note that, for the sake of space, we do not include the details of memory consumption, but they follow a similar pattern. We can conclude that in all scenarios there is some degree of optimization, achieved through the use of spare capacity that is otherwise unused. For very large clusters, or small problems, it may be interesting to have a limit on the number of devices participating, by ordering a given number by capacity and using only the adequate number of devices.

## 5. Conclusions

In this paper, we presented our approach to gathering computational power from different IoT devices to process data for multimedia applications. We created a framework that allows the dynamic creation of coalitions of devices that use their spare resources in a joint effort to provide a service with a given quality level. This implies that the devices are prepared to join their efforts with others, without compromising their original functionality. Through the application of resource reservation techniques and the flexibility of Elixir, we were able to create a framework to enable the predefined outcome. The framework is totally customizable, and QoS dimensions can be easily added or removed, given their relevance in the application scenario. The overhead added by this framework is, in our opinion, low, and the counterpoint is a promising increase in computational power. In the future, we hope to adapt streaming algorithms to use the IoT cluster we developed in a transparent and efficient way and study the use of hardware acceleration features, not exclusively the power provided by the CPU.

## Figures and Tables

**Table 3:** Cluster setup.

Device	Description	Total RAM	Available CPU (%)	Available Memory (MB)
$d_{1}$	RPi 3 B+	1.0 GB	47	350
$d_{2}$	RPi 3 B+	1.0 GB	65	835
$d_{3}$	RPi Zero W	0.5 GB	34	126
$d_{4}$	RPi 3 A+	0.5 GB	53	277
$d_{5}$	RPi Zero W	0.5 GB	15	35

**Table 4:** Services requested.

Service Name	Device Requesting	Service Description
$S_{1}$	$d_{1}$	FHD video and very high audio quality
$S_{2}$	$d_{2}$	SD video and medium audio quality
$S_{3}$	$d_{1}$	FHD video and very high audio quality

**Table 5:** Assignment of services to devices.

Available			Adding $S_{1}$		Adding $S_{2}$		Adding $S_{3}$	
Device	CPU	RAM	CPU	RAM	CPU	RAM	CPU	RAM
$d_{1}$	47	350	27	294	19	267	–	–
$d_{2}$	65	835	45	779	37	752	17	696
$d_{3}$	34	126	7	70	–	–	–	–
$d_{4}$	53	277	33	221	25	194	5	138
$d_{5}$	15	35	–	–	3	8	–	–

**Table 6:** Characterization of devices.

Type	Total RAM	Low		Medium		High	
		CPU	MEM	CPU	MEM	CPU	MEM
1	1 GB	8	27	15	45	20	56
2	0.5 GB	8	27	16	45	20	56
3	0.5 GB	12	27	21	45	27	56

**Table 7:** Devices in the simulation.

Device	Type	Available RAM (MB)	Available CPU (%)
$d_{1}$	1	652	56
$d_{2}$	1	375	85
$d_{3}$	2	458	47
$d_{4}$	3	216	61
$d_{5}$	2	127	36
$d_{6}$	3	89	15
$d_{7}$	3	127	26
$d_{8}$	1	450	64
$d_{9}$	1	784	82
$d_{10}$	2	318	63
$d_{11}$	2	287	41
$d_{12}$	2	299	76
$d_{13}$	3	30	13
$d_{14}$	1	128	24
$d_{15}$	3	280	53

**Table 8:** Service description.

Description	Type
$S_{1}$	Low
$S_{2}$	High
$S_{3}$	Low
$S_{4}$	Medium
$S_{5}$	Medium

## Data Availability

Not applicable.
